# Mining of efficient microbial UDP-glycosyltransferases by motif evolution cross plant kingdom for application in biosynthesis of salidroside

**DOI:** 10.1038/s41598-017-00568-z

**Published:** 2017-03-28

**Authors:** Bo Fan, Tianyi Chen, Sen Zhang, Bin Wu, Bingfang He

**Affiliations:** 1 College of Biotechnology and Pharmaceutical Engineering, Nanjing Tech University, 30 Puzhunan Road, 211816 Nanjing, China; 2 School of pharmaceutical sciences, Nanjing Tech University, 30 Puzhunan Road, 211816 Nanjing, China; 3 Jiangsu National Synergetic Innovation Center for Advanced Materials, 30 Puzhunan road, Nanjing, 211816 Jiangsu China; 4 Jiangsu Collaboration Innovation Center of Chinese Medical Resources Industrialization, Nanjing University of Chinese Medicine, 138 Xianlin Road, 210023 Nanjing, China

## Abstract

The plant kingdom provides a large resource of natural products and various related enzymes are analyzed. The high catalytic activity and easy genetically modification of microbial enzymes would be beneficial for synthesis of natural products. But the identification of functional genes of target enzymes is time consuming and hampered by many contingencies. The potential to mine microbe-derived glycosyltransferases (GTs) cross the plant kingdom was assessed based on alignment and evolution of the full sequences and key motifs of target enzymes, such as *Rhodiola*-derived UDP-glycosyltransferase (UGT73B6) using in salidroside synthesis. The GTs from *Bacillus licheniformis* ZSP01 with high PSPG motif similarity were speculated to catalyze the synthesis of salidroside. The UGT_BL_1, which had similarity (61.4%) PSPG motif to UGT73B6, displayed efficient activity and similar regioselectivity. Highly efficient glycosylation of tyrosol (1 g/L) was obtained by using engineered *E*. *coli* harboring UGT_BL_1 gene, which generated 1.04 g/L salidroside and 0.99 g/L icariside D2. All glycosides were secreted into the culture medium and beneficial for downstream purification. It was the first report on the genome mining of UGTs from microorganisms cross the plant kingdom. The mining approach may have broader applications in the selection of efficient candidate for making high-value natural products.

## Introduction

Natural products are a major source of current clinical drugs and are a substantial resource for the discovery of new drugs^1^. Plants are abundant in secondary metabolites. The development of plant-derived medicines has initiated from the acquisition of pure bioactive components from herbs. Unfortunately, these components are hard to collect and purify owing to their low content in medicinal plants. For this reason, an intensive search for alternative methods that can enhance the production of natural products is necessary^2^.

Many secondary metabolites exist as glycosides^3^ and exert several pharmacological properties, such as estrogenic^4^, antibacterial^5^, antiviral^6^, anti-inflammatory^7^, endocrinological^8^ and anti-cancer^9^. Glycosylation is one of the key mechanism in determining the chemical diversity of natural products. At present, both glycosidase and glycosyltransferase can be used in the synthesis of glycosides. However, the hydrolysis activity of glycosidases commonly result in low glycoside yields^10^. Several strategies, such as protein engineering and solvent engineering, have been applied to enhance the glycosylation potential of natural glycosidases^11,12^. From a practicality standpoint, beside microbial glycosidase, microbial glycosyltransferase is a favorable candidate for glycosylation because of the low cost, the high reaction yields and high concentrations of reactants and easy genetically improvement. Glycosyltransferase (GT, EC 2.4.x.y), which belong to the transferase family, has been identified in all of the three kingdoms (plants, animals and microbes). In comparison to microorganisms, plants have more GT genes in their genome, for example, there are 120 and 96 UDP-GTs in *Arabidopsis thaliana* and *Cicer arietinum*, respectively^13,14^. These enzymes are involved in the modification of secondary metabolites, such as phytohormones and xenobiotics, through the transfer of sugar moieties from an activated nucleotide molecule to a wide range of acceptors^15,16^. The available extensive database of GTs from diverse plant families is critical to determining the characteristics of target enzymes.

The synthetic biology provides a significant driving force in the synthesis of natural products through the assembly of plant-derived biosynthetic pathway. Although, the synthetic biology has received much attention in making high-value nature products, most of them are hampered with low efficiency^17^. One bottleneck is that plant-derived enzymes are sometimes unsuccessfully expressed or remain lower activity in the engineering bacteria. Fortunately, the effective microbe-derived enzymes could provide a valid sustain for creating hybrid metabolic pathway of high-value chemicals^18^. Although the available microorganism genome databases are increasing, previous gene identification methods of target proteins were mainly focused on biochemical studies and characterizations, and genome mining were largely based on the alignment of enzymes from similar genera and species. We propose that the mining of novel microbial biocatalysts for the production of natural products should be addressed through phylogenetic analysis of the complete sequences and key amino acid sequence motifs of enzymes generating secondary metabolites cross the plant kingdom. Phylogenetic tree analysis can be useful to deduce the structure-function relationship of the predicted GTs and to further assist in functional analysis^13,19^.

This report addresses the challenge: genome mining of microbe-derived GTs possessing similar characteristics to those GTs from the plant kingdom. On the basis of alignment and analysis of complete sequences or conserved signature motifs, *Bacillus licheniformis* derived GTs with high PSPG motif similarity to UGT73B6 were mined according to the phylogenetic analysis. Their catalytic products were detected and identified with *Rhodiola*-derived GT. To our knowledge, there are no reports on the synthesis of salidroside by microbial GTs. The mining approach cross the plant kingdom should assist in the understanding of functional genes, predicting the structure-function relationship of the target enzymes and the precise selection of candidate genes from microbes for the efficient biosynthesis of important natural products.

## Results

### Evolution of the full sequence and PGPG motif of GTs

**Figure 1:**
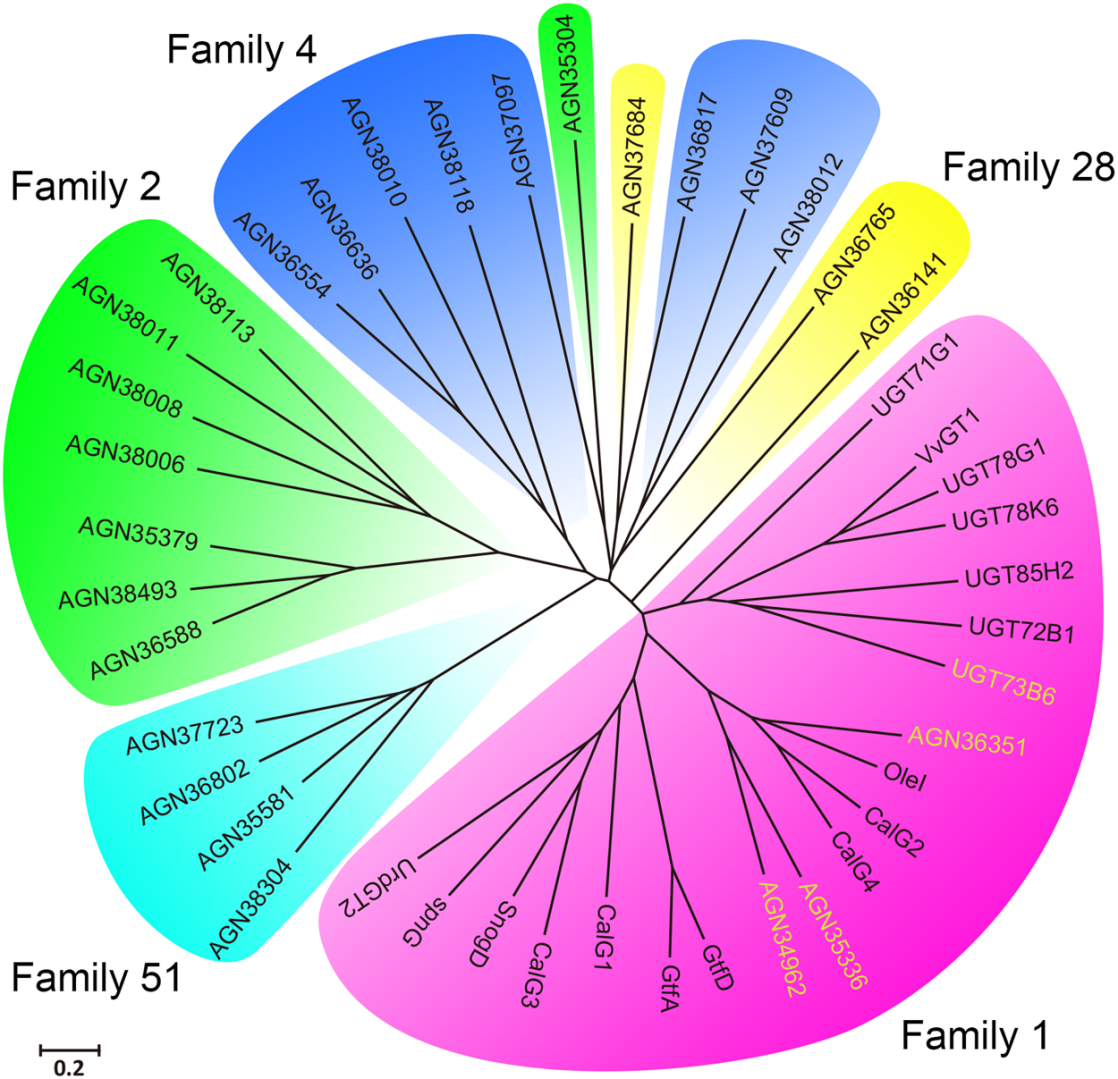
The phylogenetic tree of glycosyltransferases full sequences. The glycosyltransferases (GTs) sequence obtained from the NCBI database, including plant GTs(UGT73B6, UGT85H2, UGT72B1, UGT78K6, UGT78G1, VvGT1, UGT71G1), microorganism GTs(OleI, CalG4, CalG2, GtfA, GtfB, GtfD, spnG, UrdGT2, CalG1, CalG3, SnogD and GTs from Bacillus licheniformis 9945A).

Glycosylation play an important role in synthesis of many natural products. It is primarily catalyzed by a class of enzymes known as GTs, which belong to a large transferase family. To search efficient GTs for the biosynthesis of natural products, a phylogenetic tree based on the full amino acid sequences of selected GTs was constructed (Fig. 1). In these GTs, seven well characterized plant-derived family 1 GTs for natural products synthesis are include, such as UGT73B6 (*Rhodiola*), UGT72B1 (*Arabidopsis thaliana*), UGT78K6 (*Clitoria ternatea*), UGT71G1/UGT85H2/UGT78G1 (*Medicago truncatula*) and VvGT1 (*Vitis vinifera*). Their genetic information can be a reference for speculating the structure and function of other enzymes. Other ten well-documented microbial family 1 GTs are from *Actinomyces* genus (*Streptomyces antibioticus*: OleI, *Micromonospora echinospora*: CalG1/CalG2/CalG3/CalG4, *Amycolatopsis orientalis*: GtfA/GtfD, *Saccharopolyspora spinosa*: SpnG, *Streptomyces nogalater*: SnogD, *Streptomyces fradiae*: UrdGT2). The rest twenty six GTs are from *Bacillus licheniformis* 9945A, including three family 1 GTs, eight family 2 GTs, eight family 4 GTs, three family 28 GTs and four family 51 GTs. The phylogenetic tree comprises five main clusters corresponding to the five GT families (family 1, family 2, family 4, family 28, family 51). As an exception, the AGN35304 from family 2 and the AGN37684 from family 28 are inserted into the cluster of family 4 GTs. Meanwhile, only low homology and similarity can be observed in the phylogenetic tree of full sequence.

**Figure 2:**
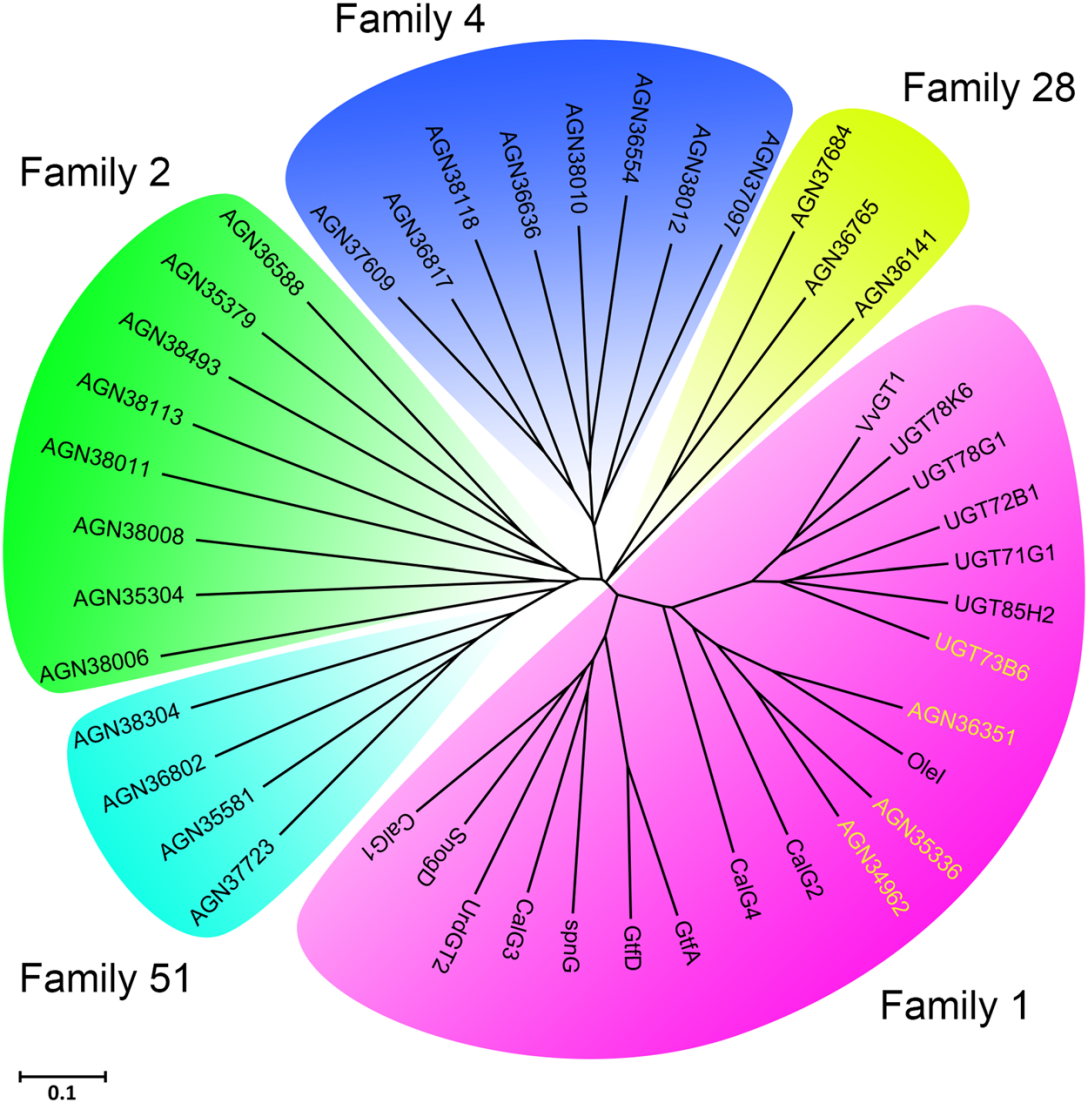
The phylogenetic tree of glycosyltransferases PSPG Motifs.

The Plant Secondary Product Glycosyltransferase (PSPG) box is a highly conserved motif in plant-derived family 1 GTs. A phylogenetic tree based on the PSPG motif region of the selected GTs was generated (Fig. 2). The family 1 GTs are still in one cluster just as the full sequence phylogenetic tree shows. In addition, the AGN35304 and the AGN37684 are classified as family 2 and family 28 GT, respectively. Since the family 1 GTs are classified similarly in shape of phylogenetic trees based on the full sequences and the PSPG motifs, they showed obviously higher homology and similarity.

### Genome mining of microbial GTs on the basis of phylogenetic analysis cross the plant kingdom

**Figure 3:**
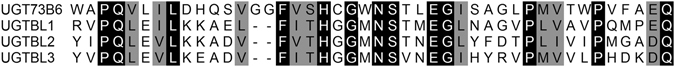
Motif sequence alignment of UGT_BL_1, UGT_BL_2, UGT_BL_3 and UGT73B6.

We have previously isolated bacterium *Bacillus licheniformis* ZSP01 on the basis of its ability to efficiently glycosylate coumarin and isoflavone, which are important secondary metabolites in the plant^20,21^. In the present study, we analyzed GTs from typical strain *Bacillus licheniformis* 9945A and many GT genes belong to several main GT families from various plants and some microorganisms, by bioinformatics approaches. In the phylogenetic tree based on the PSPG motif region, plant-derevied family 1 GTs and GTs from 9945A were classified into the same area. Moreover, according to phylogenetic analysis, three UGTs (AGN36351, AGN35336, AGN34962) with (61.4%, 59.1%, 54.5%) PSPG motif similarity to UGT73B6 were observed (Table 1). Based on the information of these GTs, three putative GT genes, *ugt*BL1, *ugt*BL2 and *ugt*BL3 (corresponding to AGN36351, AGN35336 and AGN34962 from *Bacillus licheniformis* ATCC 9945A), were cloned from the *Bacillus licheniformis* ZSP01 and separately cloned into pET28a vector. All of these three mined genes were sequenced and submitted to GenBank (obtained the accession number: KP123426, KT946966, KT946967, respectively). Microbial UGT_BL_1 shares only 18.2% identity at the amino acid sequence level, and lower similarities of UGT_BL_2 (14.9%) and UGT_BL_3 (14.0%) are shown in comparison with UGT73B6 (Table 1). A higher identity of 43.2% and similarity of 61.4% were observed between the PSPG motif of UGT_BL_1 from *Bacillus licheniformis* ZSP01 and that of UGT73B6. Slightly lower similarities were found between the PSPG motifs of UGT_BL_2 (38.6%/59.1%) and UGT_BL_3 (38.6%/54.5%) with that of UGT73B6, which notably showed differences at 30~33 residues and 40~43 residues, respectively (Fig. 3). The PSPG motif similarity of these three mined genes cloned from ZSP01 are identical with the result of AGN36351, AGN35336 and AGN34962, proving the high conservation of PSPG motif in family 1 GTs.

**Table 1:** Glycosyltransferase from *Bacillus licheniformis* ZSP01 compared with UGT73B6 from *Rhodiola sachalinensis*.

Enzyme name	Length	Identity and similarity with the whole sequence of UGT73B6		Identity and similarity with the PSPG motif of UGT73B6	
		Identity	Similarity	Identity	Similarity
AGN36351	396 aa	18.2	28.0	43.2%	61.4%
AGN34962	404 aa	15.1	28.0	38.6%	59.1%
AGN35336	397 aa	14.2	27.5	38.6%	54.5%
UGT73B6	480 aa	100%	100%	100%	100%
UGT_BL_1	396 aa	18.2%	28.2%	43.2%	61.4%
UGT_BL_2	405 aa	14.9%	26.4%	38.6%	59.1%
UGT_BL_3	397 aa	14.0%	27.3%	38.6%	54.5%

### Functional validation of the mined microbial GTs

To study the function of the putative proteins with high PSPG similarity to UGT73B6, three engineered *E*. *coli* BL21 (DE3) strains with corresponding plasmids were constructed. Their capacities for glycosylation of tyrosol were verified in the following study. The UGT_BL_1 and UGT_BL_3 were efficiently expressed in the *E*. *coli* system, whereas most of the UGT_BL_2 formed inclusion bodies and were not analyzed further (Figure S1). Both UGT_BL_1 and UGT_BL_3 exhibited glycosylation activity toward tyrosol *in vitro*. Their specific activities and regioselectivity were tested and compared with UGT73B6 (Table S3)^22,﻿23^. The UGT_BL_1, with 61.4% PSPG motif similarity to UGT73B6, showed efficient glycosylation activity and generated two glycosylated products. The two products were further purified and identified as salidroside (tyrosol hydroxyl β-glucoside) and icariside D2 (phenolic β-glucoside) through HR-MS (Figure S2) and NMR spectroscopy (Figures S3–S8). These two glycosides are identical with the products catalyzed by engineered *E*. *coli* harboring UGT73B6. UGT_BL_3, whose PSPG motif is less similar to that of UGT73B6, showed lower activity toward tyrosol (approximately 5% of UGT_BL_1 activity) and generated two types of tyrosol glucosides, thus reflecting the robust catalytic activity of microbial enzymes with high motif similarity.

The substrate specificity was further investigated. The catalytic activities of UGT_BL_1 on the tested twelve substrates were analyzed. The efficient glucosylation of aromatic alcohol and phenol catalyzed by UGTBL1 was obtained and summarized in Table 2. No glucosylation of aromatic acid and the phenol with a steric group at the ortho-position was observed.

**Table 2:** Glucosylation of aromatic alcohol and phenol catalyzed by UGT_BL_1.

Substrate	Structure	Conversion rate	Glycosylation Products distribution	
Tyrosol		90%	alcoholic glucoside	51%
			phenolic glucoside	49%
Resveratrol		96%	3-phenolic glucoside	87%
			4′-phenolic glucoside	13%
4-hydroxybenzyl alcohol		84%	alcoholic glucoside	53%
			phenolic glucoside	47%
2-hydroxybenzyl alcohol		72%	alcoholic glucoside	61%
			phenolic glucoside	39%
p-nitrophenol		75%	phenolic glucoside	100%
Cinnamic alcohol		74%	alcoholic glucoside	100%
Vanillin		86%	phenolic glucoside	100%
Ferulic acid		87%	phenolic glucoside	100%
Thymol		ND	—	
Cinnamic acid		ND	—	

### Whole cell biocatalysis and excretion of glycosylation products

**Figure 4:**
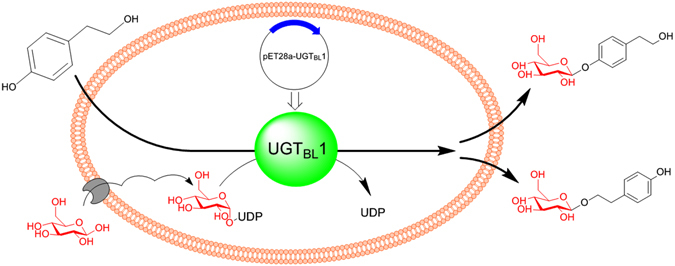
Scheme of synthesizing salidroside and icariside D2 by engineered *E*. *coli* harboring UDP-glycosyltransferase UGT_BL_1.

*In vitro* glycosylation requires expensive nucleotide sugar donors, such as UDP-glucose (UDPG). To avoid using the expensive UDPG, the BL21-UGT_BL_1 whole cell biocatalysis was conducted which can utilize glucose as the precursor and regenerate active sugar (Fig. 4). More than 90% of the tyrosol was transformed within 24 hours according to the reaction process curve (Figure S11). The strain BL21(DE3) with empty pET28a did not convert the tyrosol. Notably, the highly efficient glycosylation of tyrosol (1.0 g/L) was obtained by using whole-cell biocatalysis, resulting in 1.04 g/L salidroside, 0.99 g/L icariside D2 and a trace of residual tyrosol (Table 3). After the whole cell biotransformation, the cells were collected and ultrasonicated for the detection of substrate tyrosol and products glucosides. None of tyrosol or glucosides were observed in cells. This suggests that tyrosol could be glycosylated catalyzed by the engineered whole cell and the product glucosides extracellularly secreted into culture medium. The integrated cells were collected by centrifugation and further used in next batch biocatalysis. Many membrane proteins in *E*. *coli* involved in transporting the small molecules were also speculated by other researchers^24^. After 3 batches, the conversion yield of tyrosol maintained above 80%, and the results were attached in the Supplementary materials (Figure S10). The slight decrease of the conversion yield might be caused by the small loss of cells during the process of the cells recycle.

**Table 3:** The synthesis of salidroside: a comparison with literature results.

Production mode	product distribution		reference
	Intracellular	Extracellular	
whole cell transformation	no observed	salidroside 1.04 g/L icariside 0.99 g/L (1 g/L substrate/24 h)	this work
synthetic biology	—^a^	salidroside 0.057 g/L icariside 0.063 g/L	22
Enzymatic catalysis	—	salidroside 1.9 g/(L*d)	38
plant cell culture	salidroside 0.375 g/L	salidroside 0.06 g/L	39
plant cell transformation	salidroside 0.555 g/L	—	40
tranditional cultivation	Salidroside 1.8–6.2 g/kg^b^	—	41

^a^No reported in lecture.

^b^Dry weight.

## Discussion

Plants provide large amounts of natural products with diverse functions. But the low content of effective components in plants and their complex molecular structures make the purification and synthesis difficult. Meanwhile, many related plant-derived enzymes are hard to be expressed in microbes because of low homology. In contrast, the high catalytic activity of microbial enzymes and their ability to be genetically engineered would be beneficial for synthesis of natural products. However, the function identification of target enzymes is time consuming and hampered by traditional biochemical methods. How to rapidly discover the targeted enzymes is still a big challenge. At first, there are no reports on the syntheses or semi-syntheses of many important nature products (including salidroside) by bacteria-derived glycosyltransferase. In addition, the sequence identities or similarities of the target enzymes between different sources, such as from microorganism and from medicinal plant, are generally very low, which falls into the so-called “twilight zone” in bioinformatics, so the alignment of some key motifs closely related with active centre could make the mining more reliable. Medicinal plants provided abundant gene resources concerned with nature products. It would be favorable for mining microbe-derived GTs cross the Plant Kingdom with the help of motif evolution analysis. *Bacillus licheniformis* ATCC 9945A is the most related strain to ZSP01 on 16S rRNA and most of the GTs belong to the NDP GT-like family and the GT/glycogen phosphorylase superfamily^25–27^. As a reference, GTs from plants and other microbes for natural products synthess were selected, and all of these GTs have been well characterized. A full sequence phylogenetic tree was constructed to analyze the GT genes from Bacillus licheniformis ATCC 9945A. As a result, they were putative and classified to different GT families which was corresponding to the information in CAZY (Carbohydrate-Active EnZymes) database^28^. Family 1 UDP-GTs adopting a GT-B fold, belong to inverting UGTs, resulting in an inverted anomeric configuration in the glycosylation product β -glycoside^26^. Family 2 and family 51 reveal GT-A and lysozyme-type GTs respectively, and their 3D structures are in contrast to that of family 1^25^. Family 4 adopts GT-B structural topology similarly to family 1, but yields stereochemical glycosylation products, resulting in α-glycoside^25,26^. Family 28 is distantly related to family 1 according to the CAZY database (carbohydrate-active enzymes database)^28^. In the phylogenetic tree of full sequence, family 1 cluster comprised all the GTs from plants, other microbes and *Bacillus licheniformis* 9945A. But low homology and similarity (no more than 30%) were observed between family 1 GT members. The sequence identities of target proteins are in the so-called twilight zone, thus it is difficult to accurately predict the target protein structures and functions through bioinformatics methods. In addition, the irregular classification of the two GTs (AGN35304 and AGN37684) shown in the phylogenetic tree of full sequence may also result from the low similarity.

Research on many enzymes related to natural product synthesis shows that: their genes usually process similar conserved motifs. Some motifs play an important role in maintaining the function of proteins. So we also introduced a phylogenetic tree based on the PSPG box of the selected GTs to futher study the motif evolution of the GTs. The N-terminal domain (NTD) of UGTs, containing a conserved histidine residue and localized close to both bound sugar donor and acceptor molecules, has been demonstrated to play a crucial role in catalysis^29,30^. However, the enzymes of UDP- GT family 1 show increased variability in their N-terminal regions^31,32^. Fortunately, a highly conserved motif near their C-terminus, the PSPG box, has been identified^29^. It is an essential component of plant secondary metabolite UGTs, and specific conserved residues in the PSPG motif constitute the donor-sugar binding pockets^16,33^. In the phylogenetic tree based on the PSPG box, not only the family 1 GTs were classified into one cluster, but the other GTs were split into four clusters in accordance with their families. The result indicated that the classification based on motif evolution showed a higher correlation to their functions. Meanwhile, higher similarities were observed between members in the new phylogenetic tree. We propose that the residues of the PSPG motif not only are functionally important to donor specificity but also affect the specificity of the sugar acceptor substrate. The *Rhodiola*-derived UGT73B6 is a typical family 1 GT catalyzing the biosynthesis of salidroside, a β-glucoside of tyrosol^34^. Based on the three GTs in B*acillus licheniformis* 9945A with high PSPG motif similarity to UGT73B6, we cloned corresponding GT genes from *Bacillus licheniformis* ZSP01. Higher identities and similarities were observed between the PSPG motif of the three UGTs from ZSP01 and that of UGT73B6, indicating that the three UGTs may process similar function to UGT73B6. Therefore, we made further study on the three UGTs to verify the synthetic potential of glucoside, such as salidroside.

The three UGTs mined out from *Bacillus licheniformis* ZSP01were expressed in *E*. *coli* BL21, whereas two of them showed obvious glycosylation activity toward tyrosol. They both generated two types of tyrosol glucosides thus reflecting the robust catalytic activity of microbial enzymes with similar key motifs. Both UGT_BL_1 and UGT73B6 generated two monoglucosides, with only slightly difference in their ratio. This result indicated that UGT_BL_1 displayed similary catalytical characteristics to the UGT73B6 of glycosylating both types of hydroxyl group of tyrosol. These results are the first report the genome mining of UGTs from microorganisms on the basis of alignment and the evolution of the PSPG motif cross the plant kingdom. To our knowledge, this is also the first evidence of the use of microorganism-derived UGT to catalyze the biosynthesis of salidroside and icariside. However, salidroside has been synthesized by fungal glycosidases, other than GT, with only 6.7% yield of salidroside^35^.

*In vitro* glycosylation requires expensive nucleotide sugar donors, such as UDP-glucose (UDPG). In this context, a whole cell transformation system using the engineered strain BL21-UGT_BL_1 was applied to avoid adding UDPG (Fig. 4). The salidroside yield and enzymatic activities were improved compared to the situation when engineered *E*. *coli* harboring UGT73B6 was used^22^. Because of microbial enzymes possess high catalytic activities and tractability for genetic engineering, highly efficient catalysis of important natural glycosides can be achieved.

Moreover, the glucosides of tyrosol are almost extracellular in the whole cell system, which is similar to the results reported by Bai *et al*.^22^. The uptake of substrates and the excretion of glycosides have also been demonstrated in the isoflavonoid glucosidation system catalyzed by engineered *E*. *coli* ^36^. Since the products, glucosides of tyrosol, were secreted into culture medium and no by-product was detected in the system, it can make downstream purification much simpler. In comparison to the traditional synthesis methods of glucosides such as plant cell culture, complicated components of the products can be avoided.

In a summary, the potential to mine microbe-derived GTs synthesizing salidroside cross the plant kingdom was assessed in this study based on the alignment and analysis of the full sequence and key motif of target enzyme. These findings should contribute to the mining or rational selection of candidate genes for the biosynthesis of natural products and derivatives. Large amounts of gene resources in plants would play an important role in mining efficient enzymes for production of natural products.

## Material and Methods

### Biological and Chemical materials

The bacterial strains and plasmids used in this study are summarized in Table S1. All of the PCR primers are listed in Supplementary Table S2. UDP-glucose (UDPG) was obtained from Sigma Chemical Co. (St. Louis, MO, USA). HPLC-grade methanol was purchased from Jiangsu Hanbang Chemical Co. (Nanjing, jiangsu, China). Other solvents and reagents were of analytical grade from commercial sources.

### Mining GTs in *Bacillus licheniformis* for the synthesis of salidroside

The strain ZSP01 was isolated from chemical-contaminated soil, and was identified to share same genus and species with *Bacillus licheniformis* ATCC 9945A based on 16S rRNA gene sequence. *Bacillus licheniformis* ATCC 9945A is a typical strain for which full sequence data are available in GenBank (access No. CP005965). To mine the putative tyrosol GTs, the amino acid sequences of GTs from *Bacillus licheniformis* ATCC 9945A were downloaded from the National Center for Biotechnology Information (NCBI) database. The list of the GT accession numbers could be found in the Carbohydrate-Active EnZymes database (CAZy). The amino acid sequences of other related GTs from different speices were exported from the NCBI database. The obtained sequences were aligned with UGT73B6, and a phylogenetic tree was built. All the sequences were aligned using the ClustalX (version 2.0). Phylogenetic analysis and neighbor-joining (NJ) tree construction were performed using the MEGA (version 6.06) software. The resulting sequences most similar to UGT73B6 were selected for further analysis and used to design the primers for the genetic cloning of the GTs from *Bacillus licheniformis* ZSP01.

### Gene cloning and plasmid construction

GT genes *ugt*BL1, *ugt*BL2 and *ugt*BL3 were cloned from the total genomic DNA of *Bacillus licheniformis* ZSP01. The sequences were sequenced at GENEWIZ Inc. (Suzhou, China) and submitted to GenBank (accession number, *ugt*BL1: KP123426, *ugt*BL2: KT946966, *ugt*BL3: KT946967). The gene *ugt*BL1, *ugt*BL2 and *ugt*BL3 were ligated with pET28a and generated the plasmid pET28a-UGT_BL_1, pET28a-UGT_BL_2 and pET28a-UGT_BL_3, respectively. The plamids were separately transformed into *E*. *coli* BL21 (DE3) to yield the strains BL21-UGT_BL_1, BL21-UGT_BL_2 and BL21-UGT_BL_3. The detailed protocols for plasmid and strain constructions are described in Supplementary Materials.

### Expression of the GTs

The transformed *E*. *coli* harboring the appropriate plasmid were cultured at 37 °C in Luria-Bertani medium containing 50 μg/mL kanamycin until an OD_600_ of approximately 0.8 was reached; subsequently, GT expression was induced with 1.0 mM isopropyl 1-thio-β-D-galactopyranoside for 6–8 h at 30 °C. The cells were harvested via centrifugation at 12,000 × *g* for 10 min, washed two times with sterilized water. The *E*. *coli* cells were collected and be used in the whole cell transformation. The cells suspension can be sonicated and the expression of GTs will be analyzed with sodium dodecyl sulfate polyacrylamide gel electrophoresis (SDS-PAGE). After the cells were sonicated, the supernatant was isolated by centrifugation at 12,000 × *g* for 10 min at 4 °C. Subsequently, the GTs was purified through His-tag protein purification with standard Ni-NTA resin. The protein concentration was determined by using the Bradford method.

### Glycosylation of tyrosol ***in vitro***

A 100-μL reaction was used. The typical glycosylation of tyrosol was performed in reaction mixtures containing 50 μg/mL of purified UGT_BL_1, 2 mM tyrosol, 2 mM UDP-glucose and 50 mM Tris-HCl at pH 8.0. The reaction mixture was incubated at 30 °C for 1 h and quenched with methanol. The supernatant was obtained after centrifugation at 12,000 × *g* for 10 min and analyzed by HPLC. Other aromatic alcohol and phenol substrates were tested as the same condition.

### Batch whole cell biotransformation of tyrosol (*in vivo*)

The whole cell biotransformation of tyrosol was conducted in shaking flasks at 200 rpm at 30 °C. A typical reaction was conducted in 0.05 mol/L Na_2_HPO_4_/NaH_2_PO_4_ buffer (pH 7.5) containing glucose (20 g/L)^37^, tyrosol (1.0 g/L) and cells with cell concentrations at an OD_600_ of approximately 5. After optimization, the biotransformation reaction was scaled up to 0.2 L in a 0.5-L shaker flask. After 24 h, the whole cell biotransformation culture was centrifuged at 12,000 × *g* for 10 min. The supernatant was used for *n*-butanol extraction, and the two monoglucosides were purified. The cell pellet was resuspended in deionized water, and was followed by another round of centrifugation. Subsequently, the cell pellet was suspended in methanol and sonicated. The lysate was used for further analyzed.

### Isolation of glycosylation products of tyrosol

To separate the transformation products, the supernatant of the whole cell transformation culture was extracted three times with an equivalent volume of *n*-butanol. The extracted solutions were concentrated in a vacuum at 45 °C. The products were isolated from the concentrate with semi-preparative reversed phase HPLC. Separation was performed on a semi-preparative system (P270, Daian Elite Analytical Instruments Co., Ltd., Dalian, liaoning, China) with a column (SinoChrom ODS-BP 10 μm, 20.0 mm × 250 mm). The mobile phase comprised water/methanol (89:11, v/v), and the flow rate was 10.0 mL/min.

### Analytical method

The HPLC analysis was conducted on a Dionex P680 (Sunnyvale, CA, USA) equipped with a C18 column (Kromasil® 250 mm × 4.6 mm 5 μm, Bohus, Sweden). The column temperature and flow rate were 30 °C and 1.0 mL/min, respectively. The mobile phase was water/methanol (80:20, v/v), and detection was conducted at 275 nm. For quantification of the products, samples were diluted as required and the standard calibration curves were generated with a series of known concentrations of the standard.

High-resolution mass spectrometry (HR-MS) analysis was performed in positive ion mode on an Agilent 6520 Accurate-Mass Q-TOF LC/MS platform (Palo Alto, CA, USA).

The NMR spectra were recorded on a Bruker ARX-400 spectrometer (Bruker, Rheinstetten, Germany) using (CD_3_)_2_SO as a solvent. Tetramethylsilane (TMS) was used as the internal standard.

## Electronic supplementary material

Revised Supplementary Information
