# Author Correction: Mining of efficient microbial UDP-glycosyltransferases by motif evolution cross plant kingdom for application in biosynthesis of salidroside

**DOI:** 10.1038/s41598-017-16990-2

**Published:** 2017-12-04

**Authors:** Bo Fan, Tianyi Chen, Sen Zhang, Bin Wu, Bingfang He

**Affiliations:** 1 College of Biotechnology and Pharmaceutical Engineering, Nanjing Tech University, 30 Puzhunan Road, 211816 Nanjing, China; 2 School of pharmaceutical sciences, Nanjing Tech University, 30 Puzhunan Road, 211816 Nanjing, China; 3 Jiangsu National Synergetic Innovation Center for Advanced Materials, 30 Puzhunan road, Nanjing, 211816 Jiangsu China; 4 Jiangsu Collaboration Innovation Center of Chinese Medical Resources Industrialization, Nanjing University of Chinese Medicine, 138 Xianlin Road, 210023 Nanjing, China

*Scientific Reports* **7**:463; doi:10.1038/s41598-017-00568-z; Article published online 28 March 2017

This Article contains an error in Table 2 where the Glycosylation Products distribution percentages for 3-phenolic glucoside and 4′-phenolic glucoside have been inverted and should read 13% for 3-phenolic glucoside and 87% for 4′-phenolic glucoside.

