# Supplementary material for: Mining of efficient microbial UDP-glycosyltransferases by motif evolution cross plant kingdom for application in biosynthesis of salidroside
Source: Sci Rep. 2017 Mar 28;7:463. doi: 10.1038/s41598-017-00568-z (PMC5428655; doi:10.1038/s41598-017-00568-z)
Supplement: Supplementary file 1 — Revised Supplementary Information [file 41598_2017_568_MOESM1_ESM.doc]

**Supplementary Information**

**Mining of efficient microbial UDP-glycosyltransferases by motif evolution cross plant kingdom for application in biosynthesis of salidroside**

Bo Fan^1†^ , Tianyi Chen^2†^ , Sen Zhang^4^ , Bin Wu^1,3^ and Bingfang He^1,2,3^*

**Supplementary Methods**

**Construction of strains and plasmids.**

Bacterial strains and plasmids are summarized in Table S1. All the PCR primers are listed in Table S2. The genomic DNA of strain *Bacillus licheniformis* ZSP01 was extracted with a Takara MiniBEST Bacterial Genomic DNA Extract Kit (Dalian, liaoning, China), according to the manufacturer’s instructions. The DNA manipulations were performed according to standard protocols.

The *ugt*BL1 gene, encoding glycoystransferases UGT_BL_1, were amplified from *Bacillus licheniformis* ZSP01 genomic DNA using primers UGT_BL_1-EcoR I-F and UGT_BL_1-Xho I-R. The PCR product was introduced into the *Eco*R I and *Xho* I sites of pET28a to generate pET28a-UGT_BL_1. The *ugt*BL2 gene, encoding glycoystransferases UGT_BL_2, were amplified from *Bacillus licheniformis* ZSP01 genomic DNA using primers UGT_BL_2-Nco I-F and UGT_BL_2-Xho I-R. The PCR product was introduced into the *Nco* I and *Xho* I sites of pET28a to generate pET28a-UGT_BL_2. The *ugt*BL3 gene, encoding glycoystransferases UGT_BL_3, were amplified from *Bacillus licheniformis* ZSP01 genomic DNA using primers UGT_BL_3-Nco I-F and UGT_BL_3-Xho I-R. The PCR product was introduced into the *Nco* I and *Xho* I sites of pET28a to generate pET28a-UGT_BL_3.

The plasmid pET28a-UGT_BL_1, pET28a-UGT_BL_2 and pET28a-UGT_BL_3 were separately transformed into E. coli BL21 (DE3) to yield the strains BL21-UGT_BL_1, BL21-UGT_BL_2 and BL21-UGT_BL_3, respectively.

**Glucosyltransferase Activity Assay.** The purified UGT_BL_1 and UGT_BL_3 proteins were used for activity assay. Protein concentrations were measured by the Bradford assay using bovine serum albumin as a standard. The 100 μL assay mixture contained 50 μg/mL of purified protein, 1 mM tyrosol, 4 mM UDP-glucose and 50 mM Tris-HCl at pH 8.0. Reactions were performed at 30 °C for 1 h, after which the samples were prepared for HPLC analysis. One unit (U) of glucosyltransferase activity was defined as the amount of enzyme that glycosylated 1 μmol of tyrosol per minute under the described conditions.

**Supplementary results**


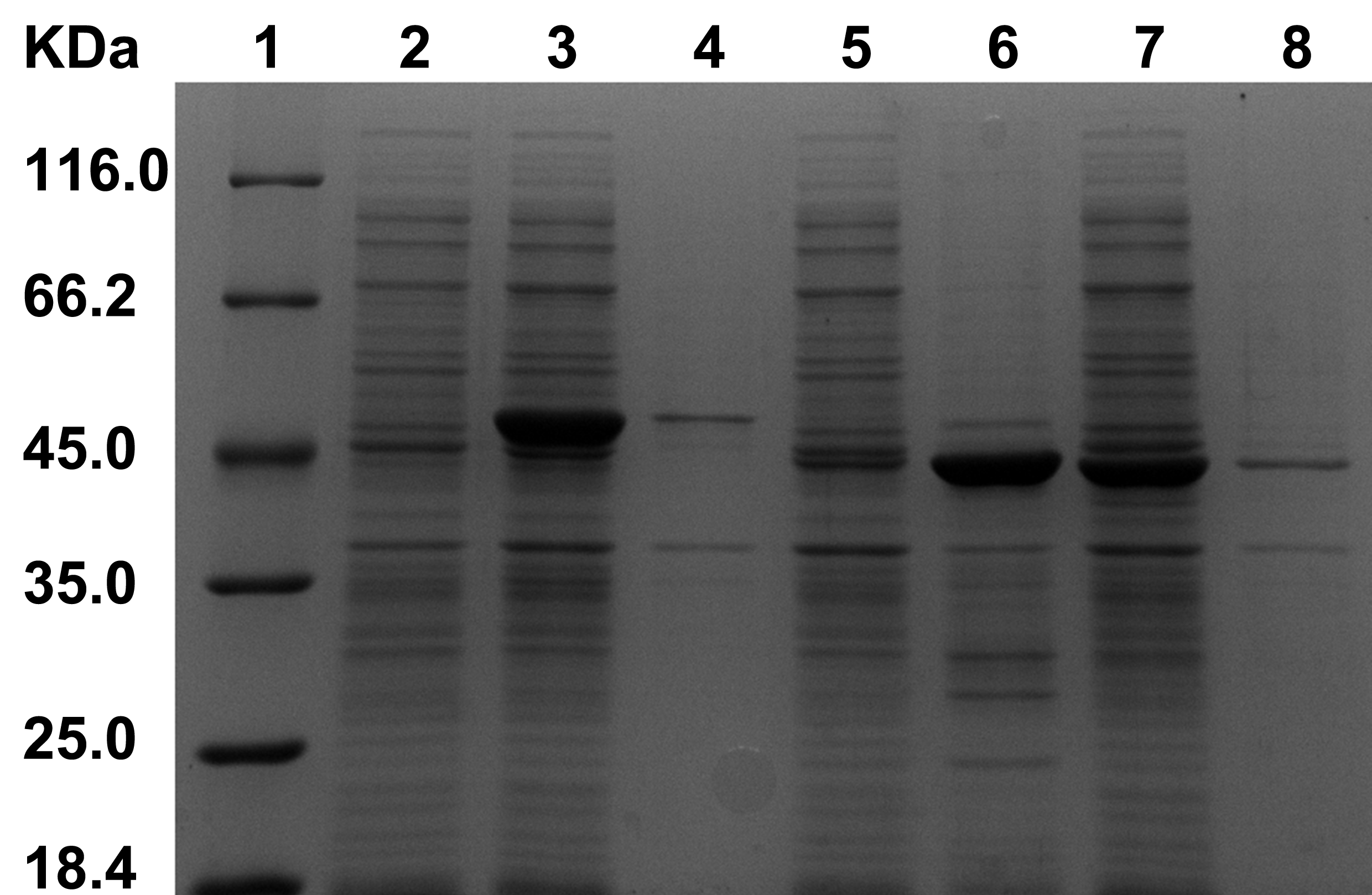


**Supplementary Figure S1. SDS-PAGE of the expression of UGT_BL_1, UGT_BL_2, UGT_BL_3. soluble faction after cell disruption insoluble.** Lane 1, protein marker; Lane 2, total protein in strain BL21-28a; Lane 3, the soluble protein in strain BL21-UGT_BL_1; Lane 4, the insoluble protein in strain BL21-UGT_BL_1; Lane 5, the soluble protein in strain BL21-UGT_BL_2; Lane 6, the insoluble protein in strain BL21-UGT_BL_2; Lane 7, the soluble protein in strain BL21-UGT_BL_3; Lane 8, the insoluble protein in strain BL21-UGT_BL_3;

**
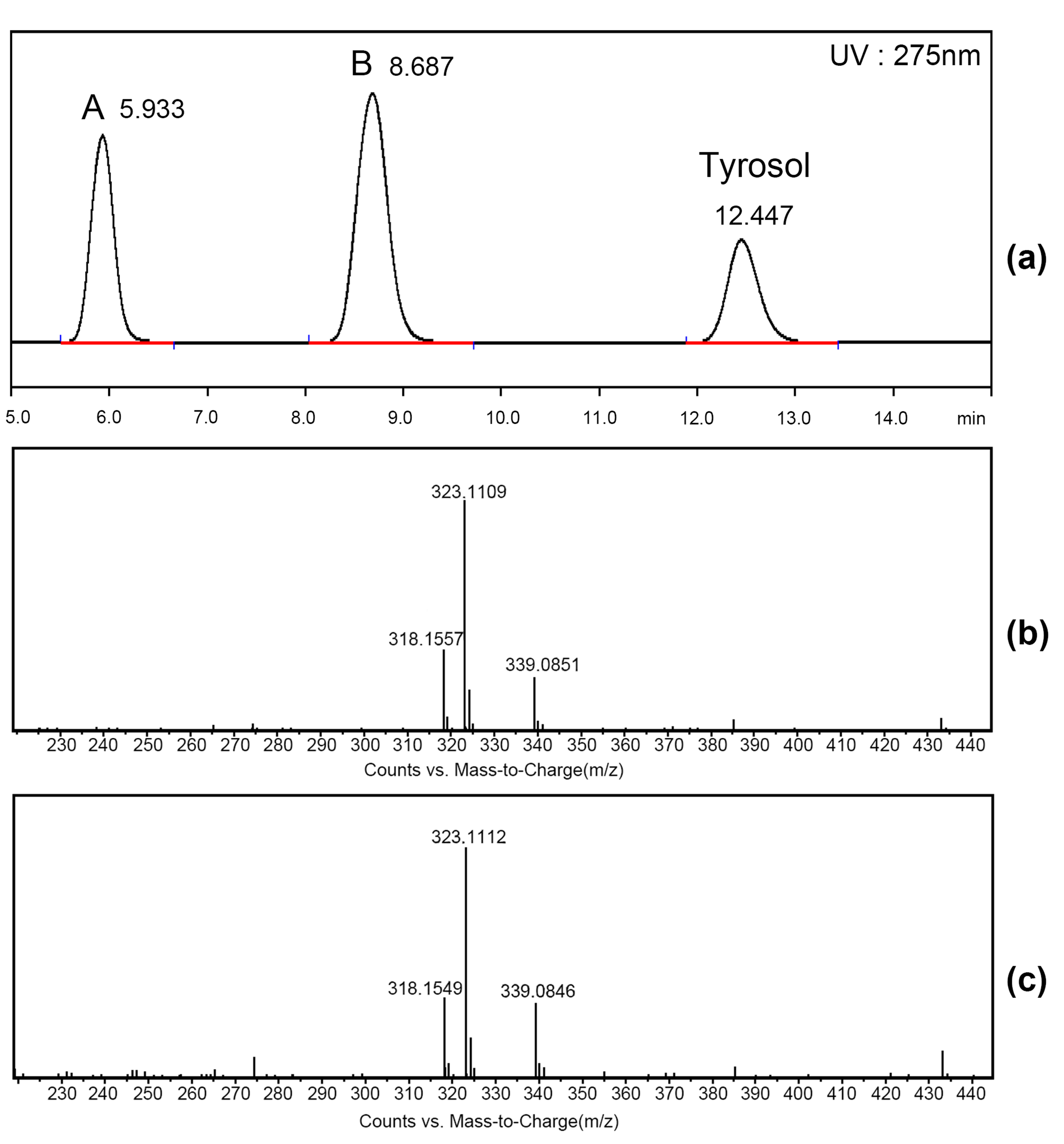
**

**Supplementary Figure S2. HPLC and HR-MS analysis of glycosylation of tyrosol by UGT_BL_1.** (a) is HPLC analysis of glycosylation of tyrosol by UGT_BL_1. (b) is HR-MS spectrogram of product A (icariside D2). (c) is HR-MS spectrogram of product B (salidroside).

**^1^H NMR (Supplementary Fig. S3) and ^13^C NMR (Supplementary Fig. S4) for salidroside.**

^1^H NMR (400 MHz, DMSO): δ 9.17 (s, 1H), 7.04 (d, *J* = 8.5 Hz, 2H), 6.72 – 6.59 (m, 2H), 4.99 (d, *J* = 4.9 Hz, 1H), 4.94 (d, *J* = 4.8 Hz, 1H), 4.90 (d, *J* = 5.0 Hz, 1H), 4.49 (t, *J* = 5.9 Hz, 1H), 4.16 (d, *J* = 7.8 Hz, 1H), 3.87 (td, *J* = 8.8, 7.0 Hz, 1H), 3.72 – 3.61 (m, 1H), 3.56 (td, *J* = 8.9, 6.6 Hz, 1H), 3.42 (td, *J* = 11.3, 5.5 Hz, 1H), 3.17 – 2.99 (m, 3H), 2.95 (td, *J* = 8.4, 4.9 Hz, 1H), 2.80 – 2.66 (m, 2H).

^13^C NMR (101 MHz, DMSO): δ 156.15, 130.32, 129.13, 115.56, 103.37, 77.42, 77.31, 73.98, 70.62, 70.47, 61.62, 35.37.


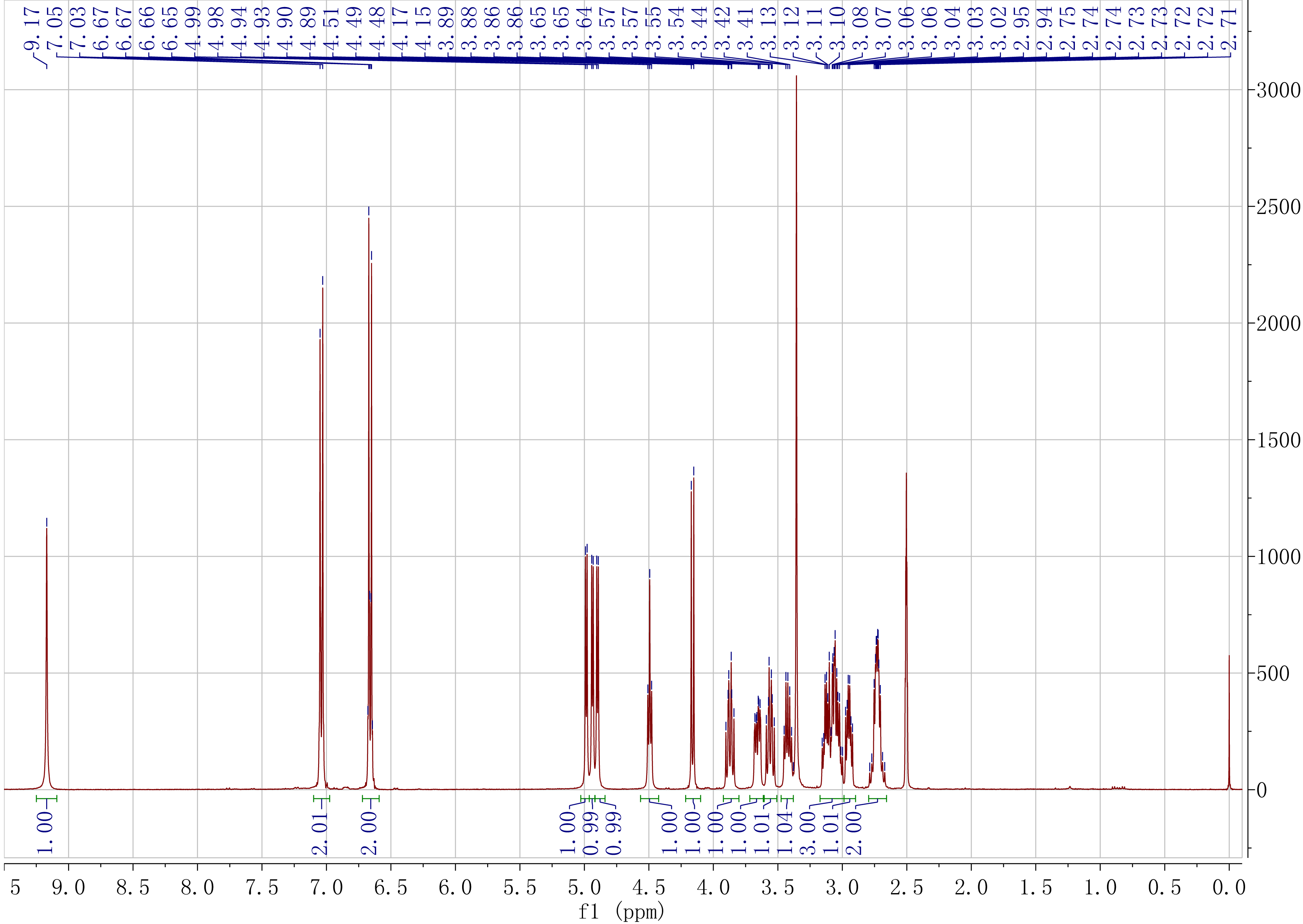


**Supplementary Figure S3. ^1^H NMR spectrum of salidroside.**

**
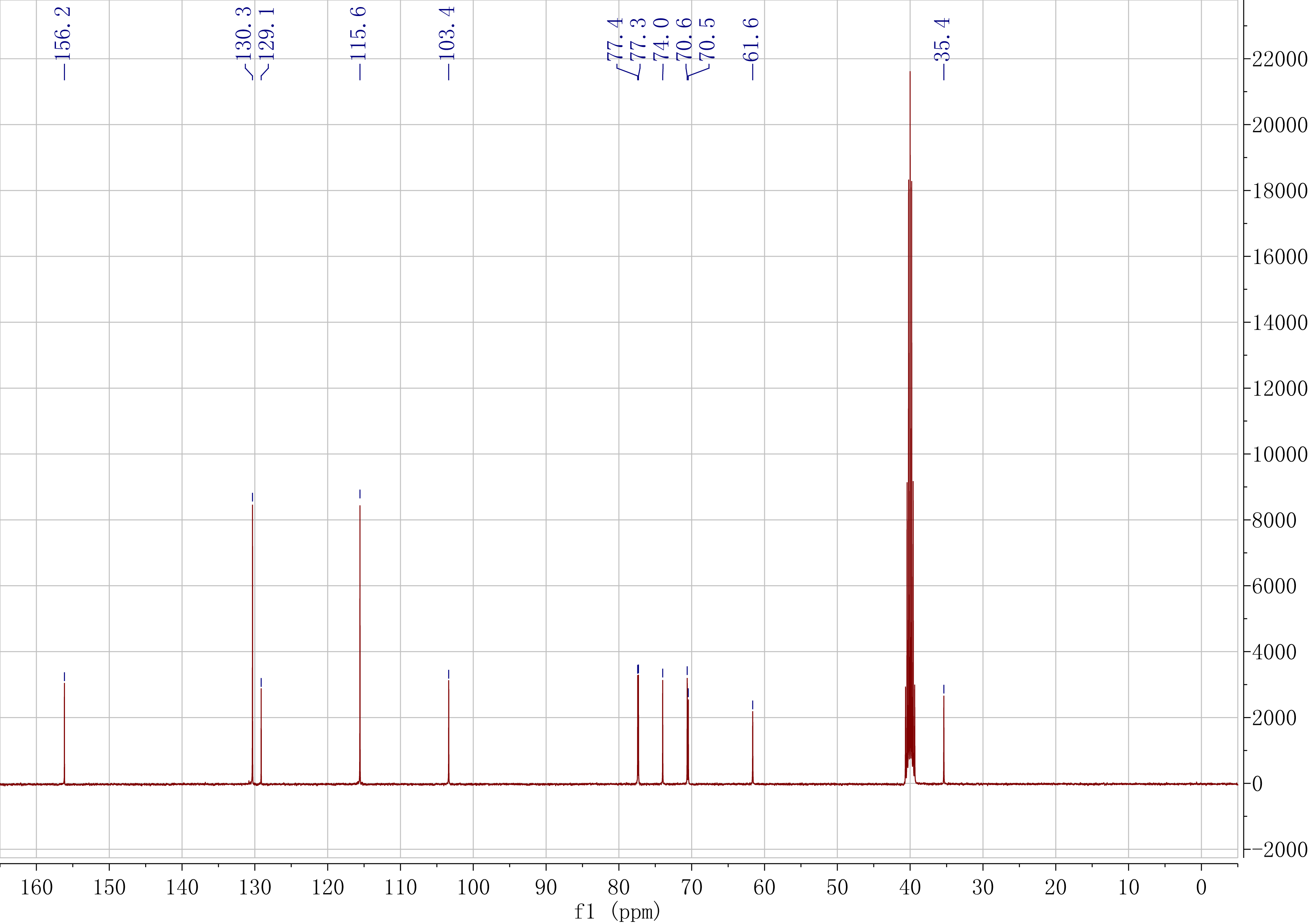
**

**Supplementary Figure S4. ^13^C NMR spectrum of salidroside.**

**^1^H NMR (Supplementary Fig. S5) and ^13^C NMR (Supplementary Fig. S6) for icariside D2.**

^1^H NMR (400 MHz, DMSO): δ 7.11 (d, *J* = 8.6 Hz, 2H), 6.93 (d, *J* = 8.6 Hz, 2H), 5.28 (d, *J* = 4.9 Hz, 1H), 5.07 (t, *J* = 6.7 Hz, 1H), 5.01 (d, *J* = 5.2 Hz, 1H), 4.79 (d, *J* = 7.4 Hz, 1H), 4.61 (t, *J* = 5.1 Hz, 1H), 4.56 (t, *J* = 5.7 Hz, 1H), 3.72 – 3.64 (m, 1H), 3.55 (dd, *J* = 12.2, 7.1 Hz, 2H), 3.45 (dt, *J* = 11.8, 6.0 Hz, 1H), 3.32 – 3.18 (m, 3H), 3.18 – 3.11 (m, 1H), 2.65 (t, *J* = 7.1 Hz, 2H).

^13^C NMR (101 MHz, DMSO): δ 156.28, 133.28, 130.20, 116.58, 101.10, 77.52, 77.17, 73.78, 70.26, 62.95, 61.24, 38.74.


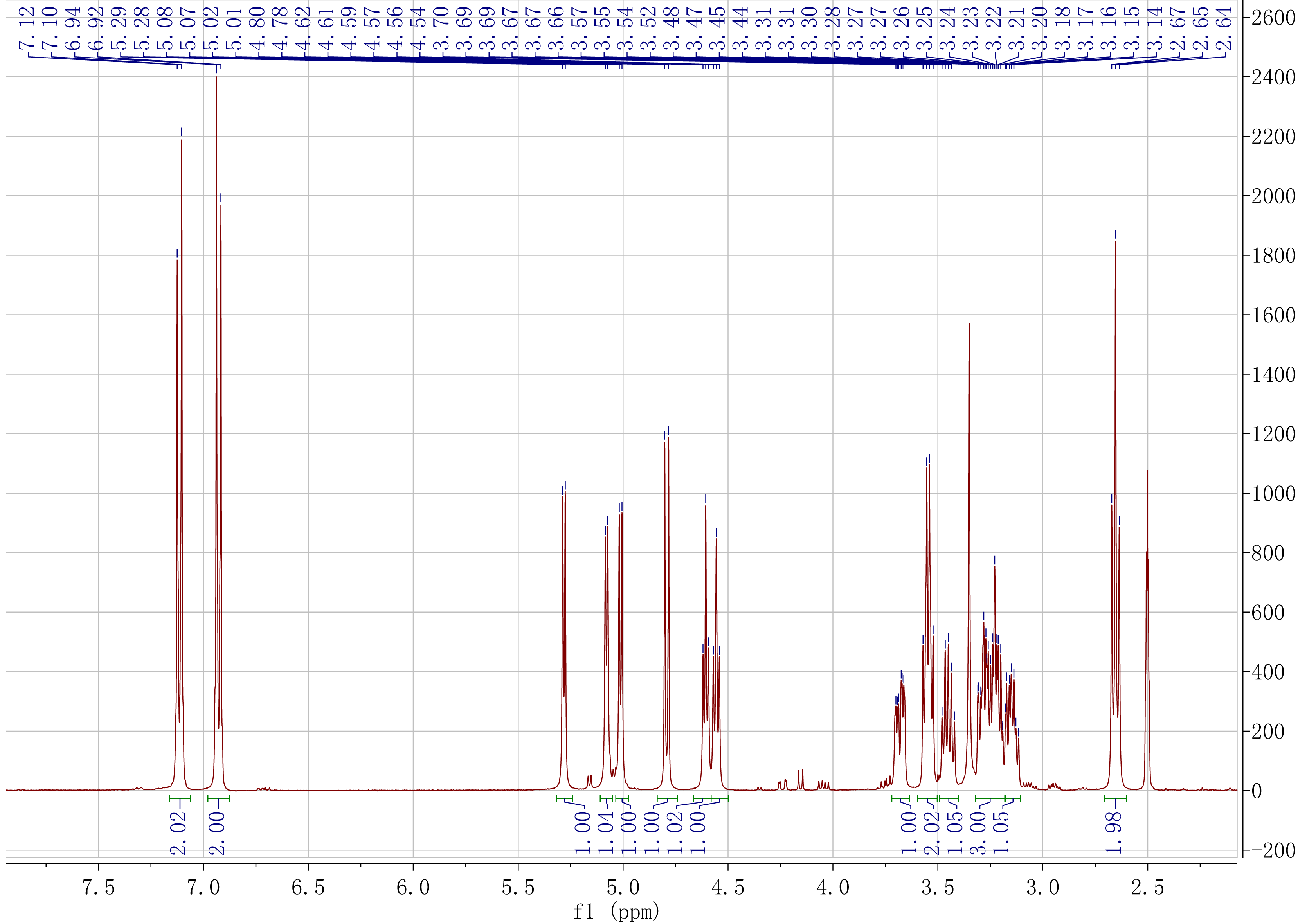


**Supplementary Figure S5. ^1^H NMR spectrum of icariside D2.**


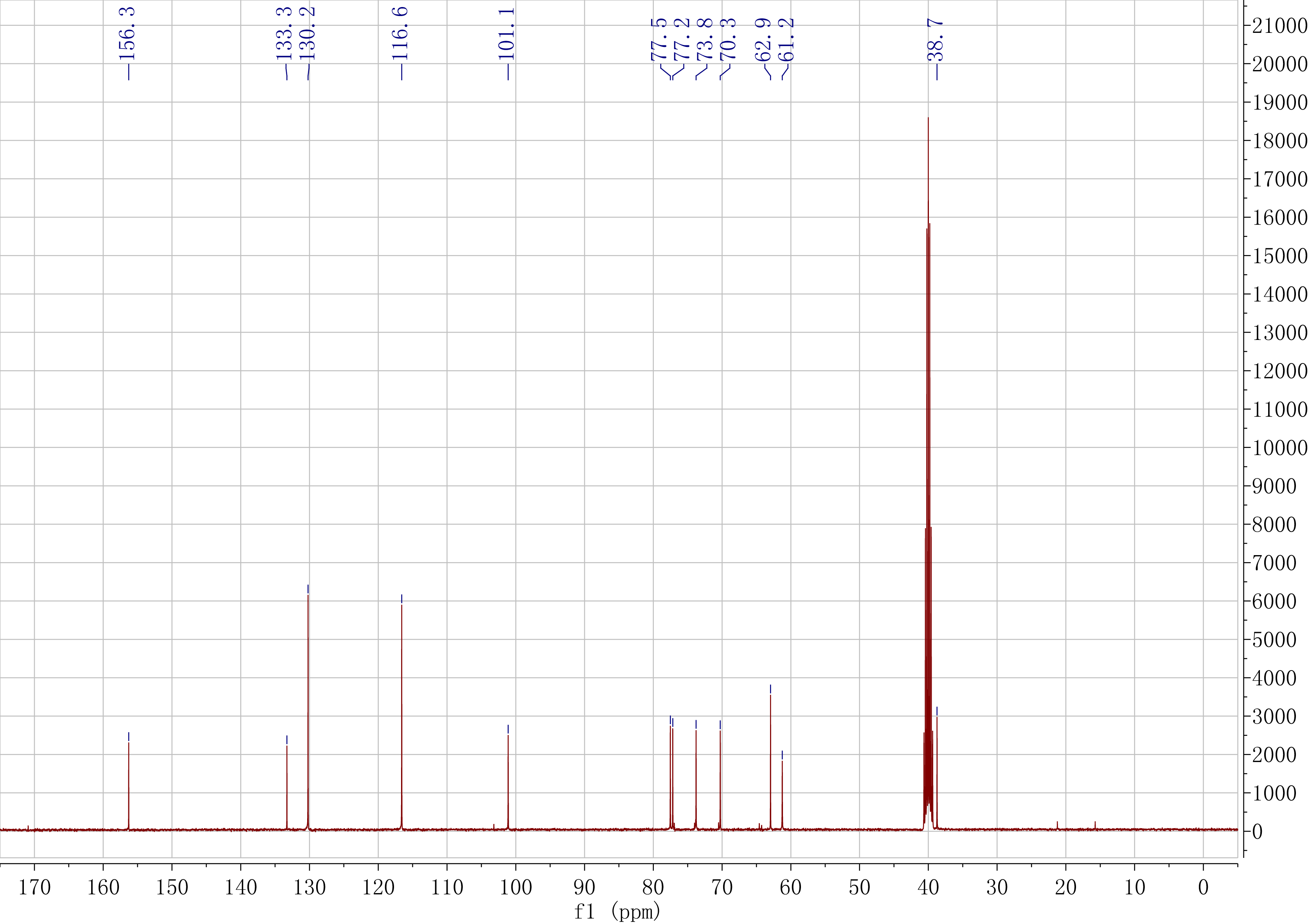


**Supplementary Figure S6. ^13^C NMR spectrum of icariside D2.**


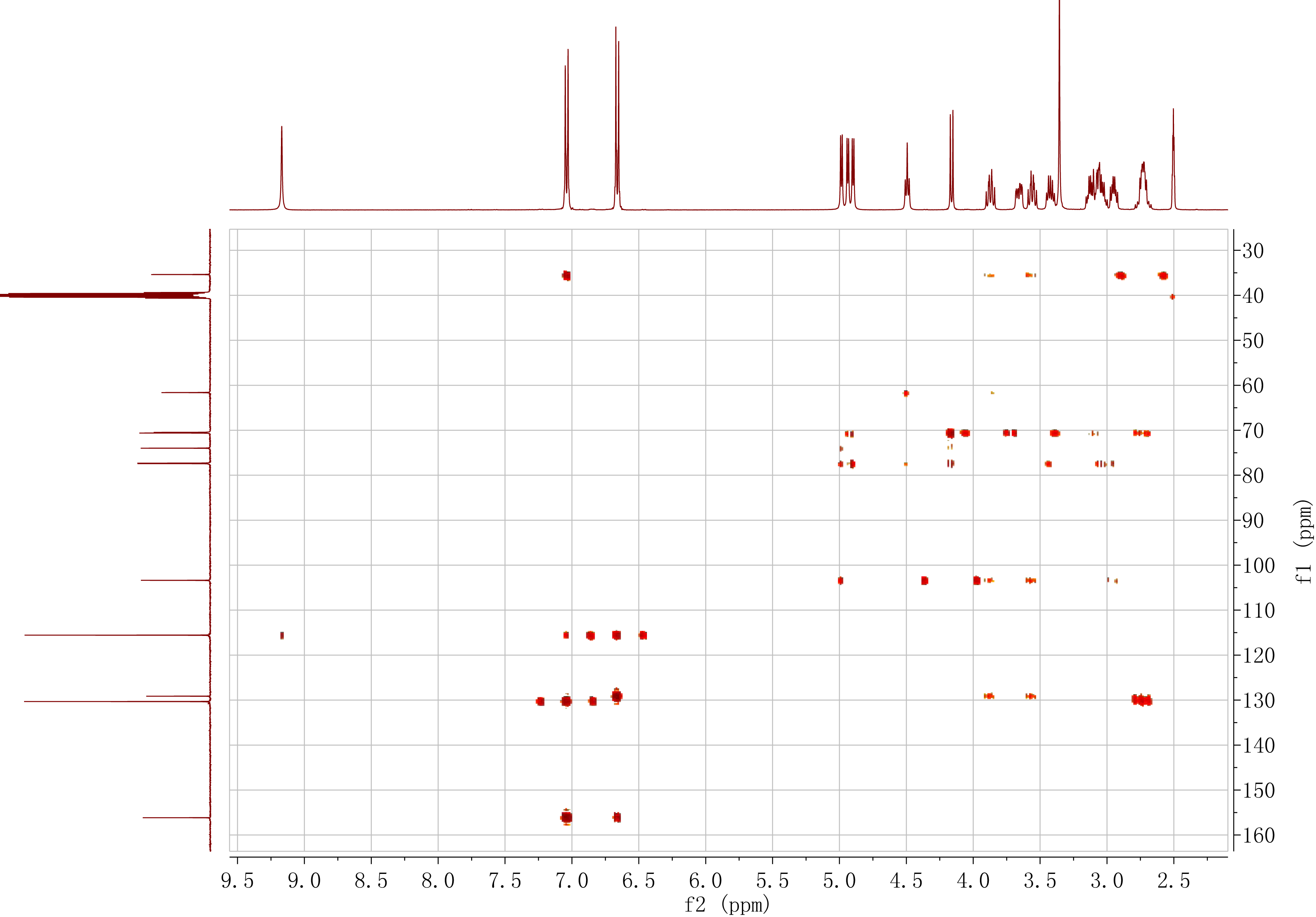


**Supplementary Figure S7. HMBC NMR spectrum of salidroside.**


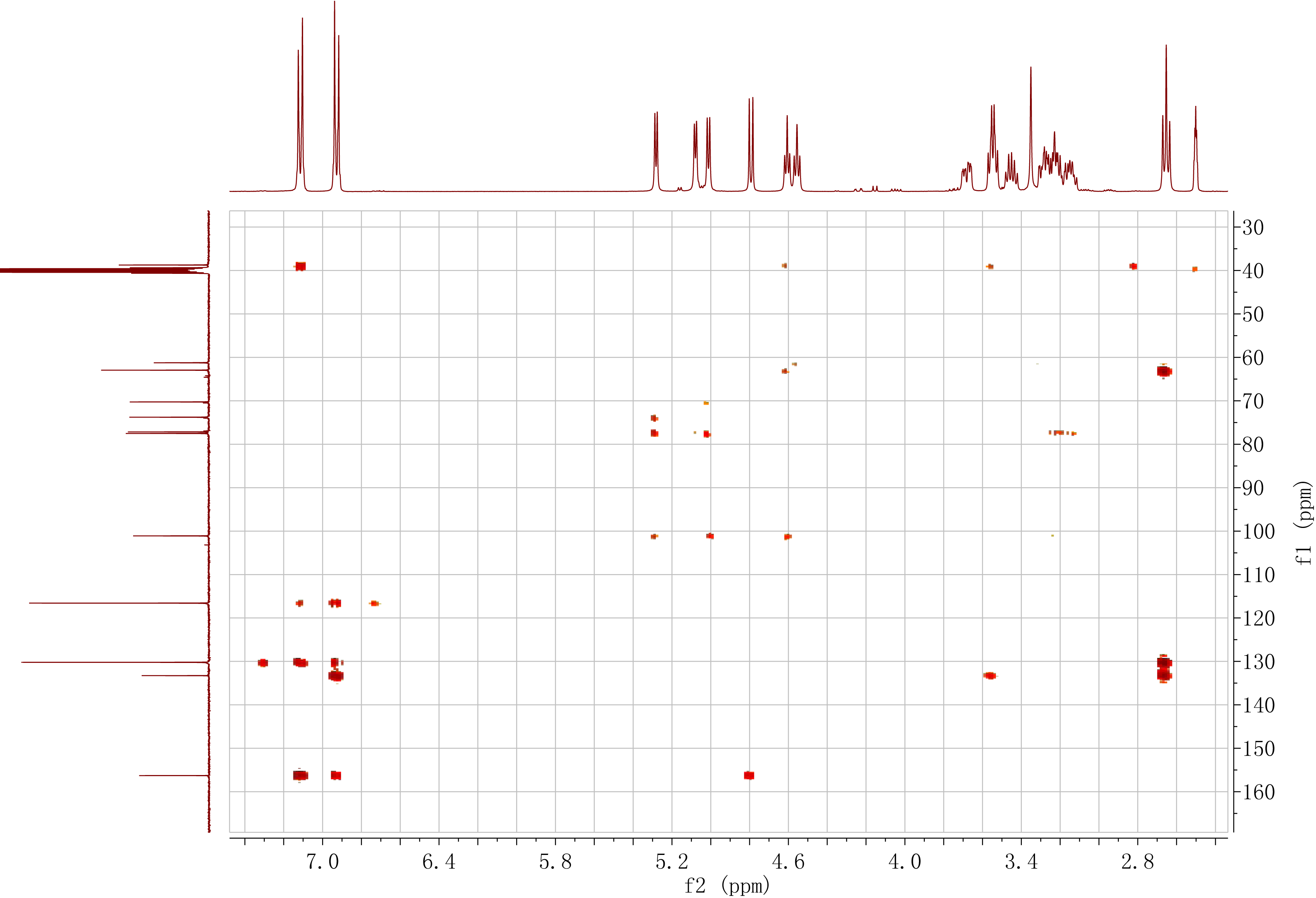


**Supplementary Figure S8. HMBC NMR spectrum of icariside D2.**


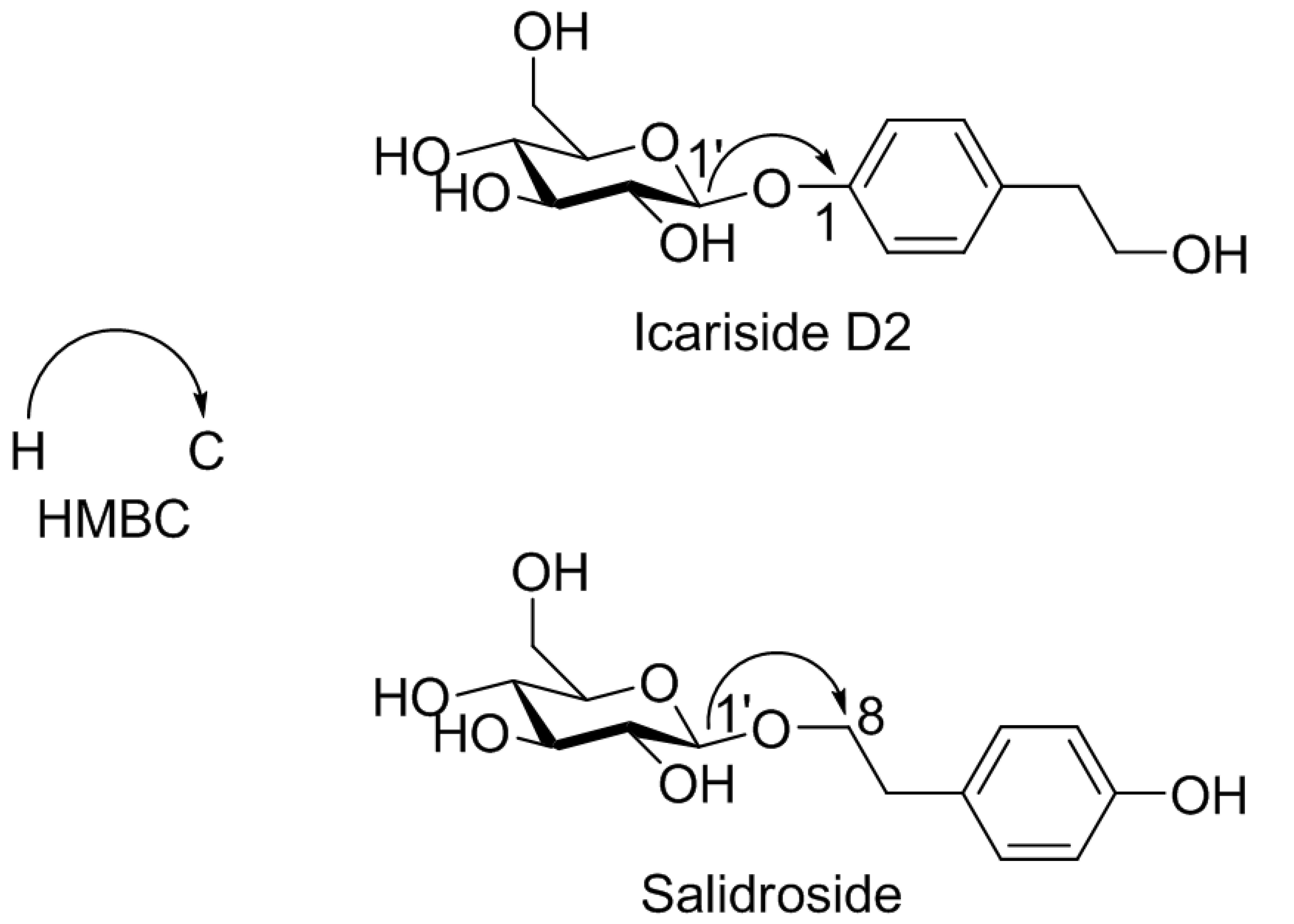


**Supplementary Figure S9. key HMBC of salidroside and icariside D2.**


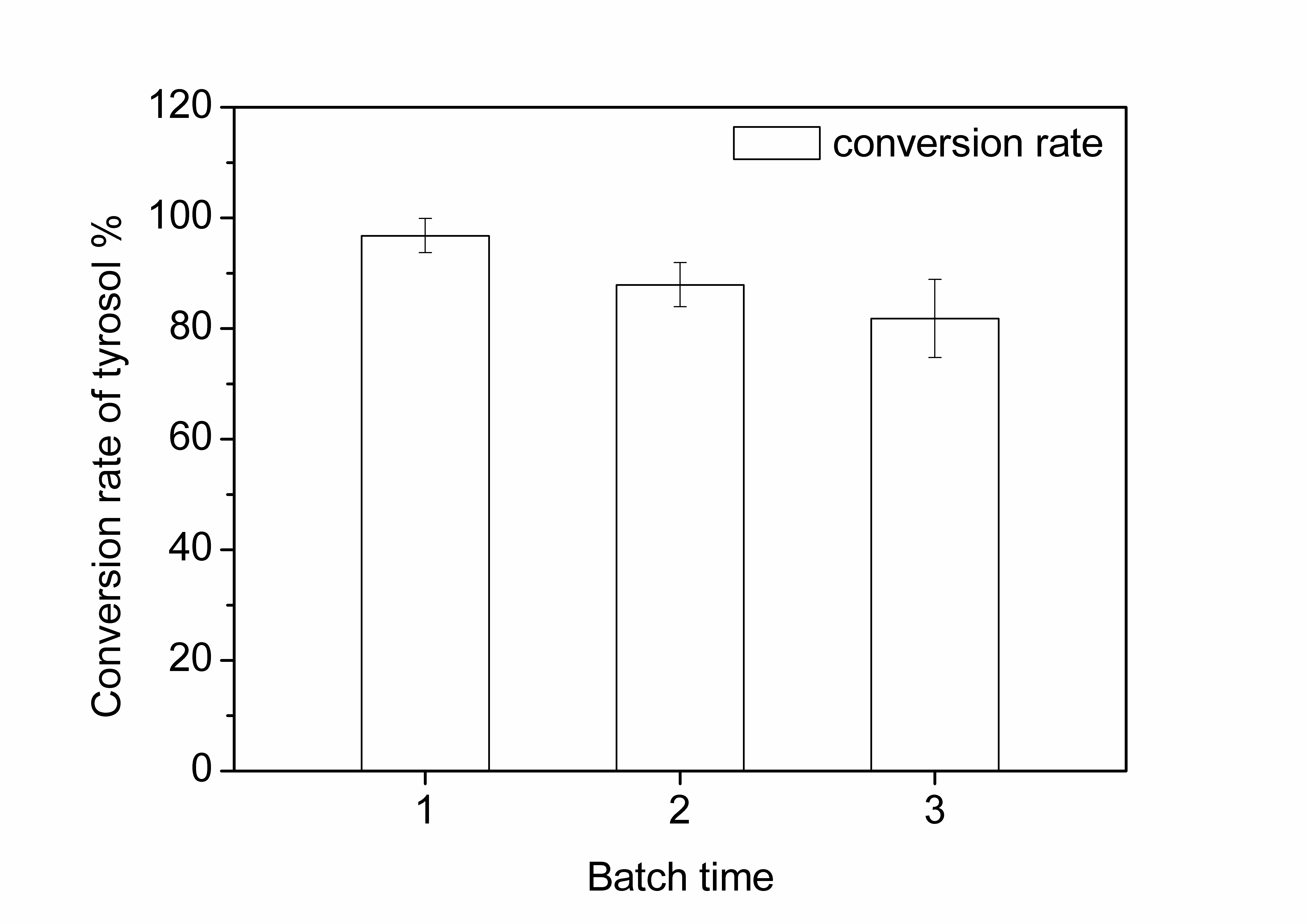


**Supplementary Figure S10. Repeated batch transformation of whole cells.**


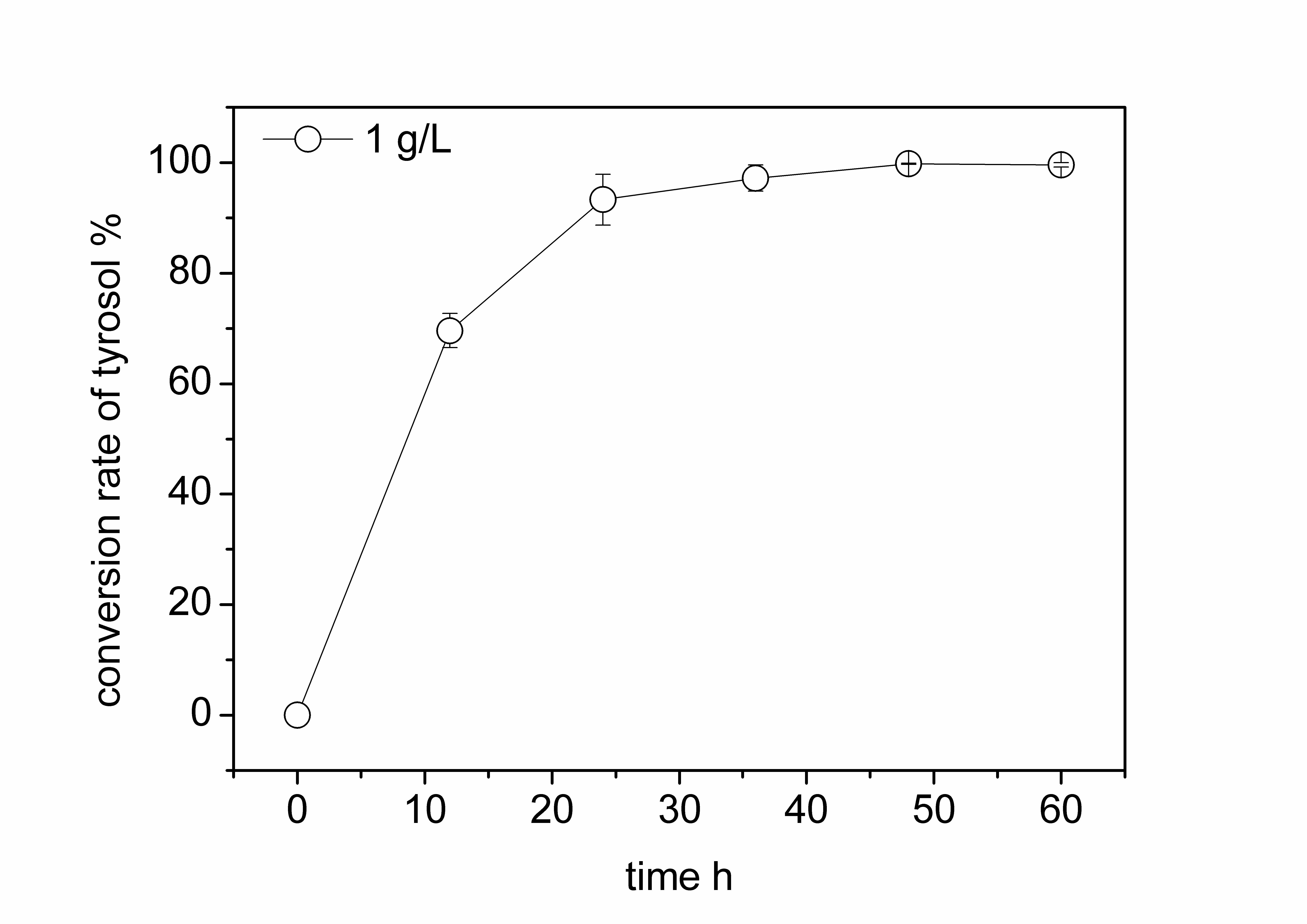


**Supplementary Figure S11. Reaction process curve of whole cell biotransformation.**

**Table S1. Strains and plasmids used in this work.**

| Strains or plasmids | Description | Source or Refs. |
| --- | --- | --- |
| Strains |  |  |
| *Bacillus licheniformis* ZSP01 | Wild-type | Lab stock |
| *E.coli* BL21 (DE3) | *F*^–^*omp*T *hsd*S(r_B_^-^, m_B_^-^) *gal* *dcm* (DE3); *E. coli* host for protein expression | Lab stock |
| BL21-28a | *E.coli* BL21 (DE3) harboring pET28a | This work |
| BL21-UGT_BL_1 | *E.coli* BL21 (DE3) harboring pET28a-UGT_BL_1 | This work |
| BL21-UGT_BL_1 | *E.coli* BL21 (DE3) harboring pET28a-UGT_BL_2 | This work |
| BL21-UGT_BL_1 | *E.coli* BL21 (DE3) harboring pET28a-UGT_BL_3 | This work |
| Plasmids |  |  |
| pET28a | ColE1 *ori*, *lac*I gene, Kan^r^, T7 protmoters | Lab stock |
| pET28a-UGT_BL_1 | *ugt*BL1 inserted between *Eco*R I/*Xho* I sites of pET28a | This work |
| pET28a-UGT_BL_1 | *ugt*BL2 inserted between *Nco* I/*Xho* I sites of pET28a | This work |
| pET28a-UGT_BL_1 | *ugt*BL3 inserted between *Nco* I/*Xho* I sites of pET28a | This work |

**Table S2. Primers used in this work (restriction sites are underlined).**

| Primer | Sequence (5’ to 3’) |
| --- | --- |
| UGT_BL_1-EcoR I-F | ATAGAATTCATGGGACATAAACATATCGCG |
| UGT_BL_1-Xho I-R | AGACTCGAGTTATTTTACTCCTGCGGGTGC |
| UGT_BL_2-Nco I-F | GCGCCATGGTGATGGGACATGTACTGATGATCA |
| UGT_BL_2-Xho I-R | TATCTCGAGACGGGGCGCTCCGT |
| UGT_BL_3-Nco I-F | GCGCCATGGCGATGAAAAATATTTTAATCGTCAATT |
| UGT_BL_3-Xho I-R | TATCTCGAGAGGCTGCAGCGATTT |

**Table S3. Specific activity and Regioselectivity of UGT_BL_1 , UGT_BL_**3 and UGT73B6.

| Enzyme | Spectific Activity to tyrosol (U/mg purified protein) | Regioselectivity (%) | |
| --- | --- | --- | --- |
|  |  | Tyrosol | Icariside D2 |
| UGT_BL_1 | 7.205±0.275 | 51.2 | 48.8 |
| UGT_BL_3 | 0.403±0.136 | 52.9 | 47.1 |
| UGT73B6 | 1.370±0.061 | 47.4 | 52.6 |
